# Coupled binding and folding of disordered SPIN N-terminal region in myeloperoxidase inhibition

**DOI:** 10.3389/fmolb.2023.1130189

**Published:** 2023-02-09

**Authors:** Yumeng Zhang, Xiaorong Liu, Jianhan Chen

**Affiliations:** Department of Chemistry, University of Massachusetts, Amherst, MA, United States

**Keywords:** instrinsically disordered region, innate immunity, atomistic simulaitons, conformational selection and induced fit, residual structures, transition path

## Abstract

Gram-positive pathogenic bacteria *Staphylococcus* express and secret staphylococcal peroxidase inhibitor (SPIN) proteins to help evade neutrophil-mediated immunity by inhibiting the activity of the main oxidative-defense player myeloperoxidase (MPO) enzyme. SPIN contains a structured 3-helix bundle C-terminal domain, which can specifically bind to MPO with high affinity, and an intrinsically disordered N-terminal domain (NTD), which folds into a structured β-hairpin and inserts itself into the active site of MPO for inhibition. Mechanistic insights of the coupled folding and binding process are needed in order to better understand how residual structures and/or conformational flexibility of NTD contribute to the different strengths of inhibition of SPIN homologs. In this work, we applied atomistic molecular dynamics simulations on two SPIN homologs, from *S. aureus* and *S. delphini*, respectively, which share high sequence identity and similarity, to explore the possible mechanistic basis for their different inhibition efficacies on human MPO. Direct simulations of the unfolding and unbinding processes at 450 K reveal that these two SPIN/MPO complexes systems follow surprisingly different mechanisms of coupled binding and folding. While coupled binding and folding of SPIN-*aureus* NTD is highly cooperative, SPIN-*delphini* NTD appears to mainly utilize a conformational selection-like mechanism. These observations are in contrast to an overwhelming prevalence of induced folding-like mechanisms for intrinsically disordered proteins that fold into helical structures upon binding. Further simulations of unbound SPIN NTDs at room temperature reveal that SPIN-*delphini* NTD has a much stronger propensity of forming β-hairpin like structures, consistent with its preference to fold and then bind. These may help explain why the inhibition strength is not well correlated with binding affinity for different SPIN homologs. Altogether, our work establishes the relationship between the residual conformational stability of SPIN-NTD and their inhibitory function, which can help us develop new strategies towards treating *Staphylococcal* infections.

## Graphical Abstract

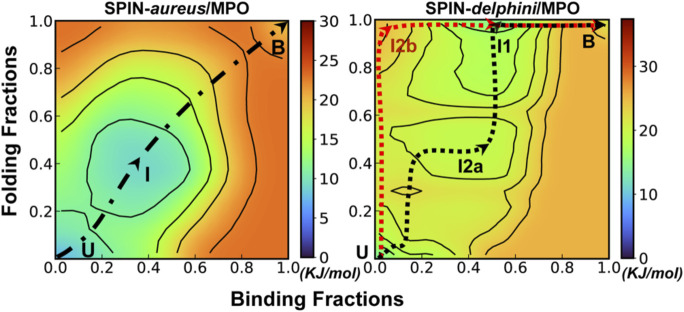

## Introduction

*Staphylococcus* is a group of gram-positive pathogenic bacteria that can lead to a broad range of infections including pneumonia and toxic shock syndrome (Lowy, 1998; Tong et al., 2015). Staphylococcal infections are becoming an increasingly severe threat to public health, with an estimate of ∼3 million cases in the United States every year and expanding incidence of antibiotic resistance (Tong et al., 2015). To defend against the invasions of *staphylococcus*, neutrophils are critical innate immune response components in hosts and serve as the first defensive line by releasing the anti-bacterium hypochlorous acid (Ploscariu et al., 2018) and other reactive oxidant species (ROS) (Rigby and DeLeo, 2012; de Jong et al., 2017). Particularly, myeloperoxidase (MPO) is one of the most abundant granule enzymes in neutrophils that can catalyze the production of ROSs from hydrogen peroxide (H_2_O_2_) to help kill the bacterium. However, *Staphylococcus* has been found to be able to evade the neutrophil-mediated innate immune defense and sometimes turn host cells into “Trojan Horses” for bacterial dissemination *in vivo* (Lambris et al., 2008; Thwaites and Gant, 2011; Kim et al., 2012; Spaan et al., 2013; Garcia et al., 2016). In particular, the bacterium can secret Staphylococcal Peroxidase INhibitor (SPIN) proteins, which bind MPO with nanomolar affinity and inhibit its enzymatic activity (de Jong et al., 2017; Ploscariu et al., 2018). SPIN consists of an intrinsically disordered N-terminal domain (NTD) and a structured 3-helix bundle C-terminal domain (CTD) (de Jong et al., 2017; de Jong et al., 2018; Ploscariu et al., 2018). The inhibitory activity requires the disordered SPIN NTD and can be largely abolished with deletion or certain mutations of the NTD region (de Jong et al., 2018). Structural studies have revealed that SPIN NTD folds into a β-hairpin and inserts itself into MPO’s active site in the complex (de Jong et al., 2018), which prevents the substrate H_2_O_2_ from accessing the catalytic heme in MPO’s active pocket. As a result, the enzyme becomes incapable of producing ROSs, thus protecting *Staphylococcus* from killing by neutrophils (de Jong et al., 2017).

Recently, multiple SPIN homologs that share high sequence identity and conformational similarity have been identified with various inhibitory capacities towards human MPO (Ploscariu et al., 2018). Interestingly, their inhibitory capacities show little correlation with their binding affinities to MPO (Ploscariu et al., 2018). For example, while SPIN-*agnetis* binds human MPO with a *K*_D_ of ∼42 nM, it has little measurable inhibitory effect on MPO activity. The implication is that, the folded SPIN CTD largely determines the binding affinity to MPO, while the disordered NTD dictates the inhibitory efficacy. Furthermore, structural studies suggest that all SPN NTD homologs likely fold into essentially the same β-hairpin conformation in the bound state (Ploscariu et al., 2018). Therefore, functional differences between SPIN homologs may be directly related to the disordered unbound state and/or the coupled binding and folding processes themselves. Specifically, two key questions are: 1) how residual structures or conformational plasticity contribute to the facile folding and binding of SPIN NTD, thus potentially impacting the inhibition strength, and 2) whether SPIN homologs show different mechanisms of coupled binding and folding.

Intrinsically disordered proteins/regions (IDPs/IDRs) like SPIN NTD are prevalent in biology and frequently play key roles in cellular regulation and signal transduction (Wright and Dyson, 1999; Dunker et al., 2001; Tsai et al., 2001; Dyson and Wright, 2005; Uversky et al., 2005; Click et al., 2010). IDPs also frequently undergo coupled binding and folding for function (Koshland, 1960; Agarwal et al., 2002; Antikainen et al., 2005; Hammes et al., 2009; Wright and Dyson, 2009; Zhou, 2010). Two classes of mechanisms have been generally invoked in studies of IDP coupled binding and folding. In so-called conformational selection-like mechanisms (Fuxreiter et al., 2004; Shammas et al., 2016; Crabtree et al., 2017; Bonetti et al., 2018; Troilo et al., 2019), residual structures in unbound state of an IDP may resemble the folded complex and serve as initial binding sites to facilitate efficient molecular recognition (that is, fold and then bind). On the other hand, an IDP could undergo rapid folding upon non-specific encountering with its target, following the so-called induced folding-like mechanism (Levy et al., 2007; Huang and Liu, 2009; Liu and Huang, 2014). Here, structural plasticity plays a more important role, such as to enable facile IDP folding on the target surface (Pontius, 1993; Oldfield et al., 2005; Turjanski et al., 2008; Gsponer and Babu, 2009; Trizac et al., 2010; Ganguly et al., 2012a; Ganguly et al., 2012b; Chu et al., 2012; Ganguly et al., 2013; Rogers et al., 2014; Liu et al., 2019). For the cases where the binding pocket is deep and rugged, induced fitting can direct the peptide to reach the spot and then fold to the energetically favored states (Sugase et al., 2007; Dosnon et al., 2015; Gianni et al., 2016; Sen and Udgaonkar, 2019; Yang et al., 2019; Toto et al., 2020). It should be noted that existing mechanistic studies have mainly involved IDPs that fold into α-helices, ordered loops or a single β-strand upon binding and that induced folding has been found to be prevalent in these IDPs (Wright and Dyson, 2009; Chen, 2012; Chen et al., 2020). SPIN NTD is notably different from these existing studies; it represents the first case study of coupled binding and folding of an IDP into a β-hairpin. Folding of β-hairpin structures involves cooperative formation of long-range contacts and has been shown to be much slower than helix-coil transitions with substantial entropy-dominant free energy barriers (Munoz et al., 1997; Klimov and Thirumalai, 2000; Chen et al., 2018). It remains unclear if SPIN-NTD will display similar mechanistic features to IDPs with simple folded structures.

In this work, we focus on two SPIN homologs, SPIN-*aureus* and SPIN-*delphini*. They share 53% sequence identity and 80% sequence similarity, and both bind to human MPO with nanomolar affinities and fold into essentially identical β-hairpin structures (Lambris et al., 2008; Thwaites and Gant, 2011; Kim et al., 2012; Spaan et al., 2013; Garcia et al., 2016). Interestingly, although SPIN-*delphini* binds to MPO ∼19 times weaker than SPIN-aureus, its half maximal inhibitory concentration (IC_50_) is only ∼6 times higher. We will mainly utilize atomistic simulations in explicit solvent to probe the conformational properties of unbound NTDs from SPIN-*aureus* and SPIN-*delphini* and to investigate their coupled binding and folding processes. We note that several coarse-grained protein models have also been recently proposed for IDPs, such as AWSEM-IDP (Wu et al., 2018), SOP-IDP (Baul et al., 2019), a modifield MARTINI model (Benayad et al., 2021) and HyRes II (Zhang et al., 2022). However, none of these models is capable of describing both folded and disordered protein states required for studies of coupled binding and folding. Instead, atomistic simulations have significantly benefited from recent advances in both GPU-enabled MD algorithms (Phillips et al., 2005; Brooks et al., 2009; Eastman et al., 2012; Gotz et al., 2012; Abraham et al., 2015; Case et al., 2017), which can provide over 100-fold acceleration compared to traditional CPU-based approaches, and accurate general-purposed protein force fields (Nerenberg et al., 2012; Piana et al., 2015; Robertson et al., 2015; Huang et al., 2017; Robustelli et al., 2018; Liu and Chen, 2019), which have been extensively rebalanced for describing both folded and disordered proteins. Simulations of temperature-driven dissociation process of two SPIN/MPO complexes at 450 K recapitulate that SPIN CTD dominates specific binding to MPO and further reveal surprising differences in coupled binding and folding of NTD of these two SPIN homologs. The binding and folding are highly cooperative for SPIN-*aureus* NTD, while SPIN-*delphini* NTD prefers to be partially folded before binding to the MPO active site. Further simulations at the room temperature show that unbound SPIN-*delphini* NTD is much more structured. These results suggest an important role of residual structures of SPIN NTD in its facile recognition and inhibition of MPO, which may help us better understand the sequence-structure-function relationship of SPIN.

## Methods

### High temperature simulations of SPIN/MPO complexes

**FIGURE 1:**
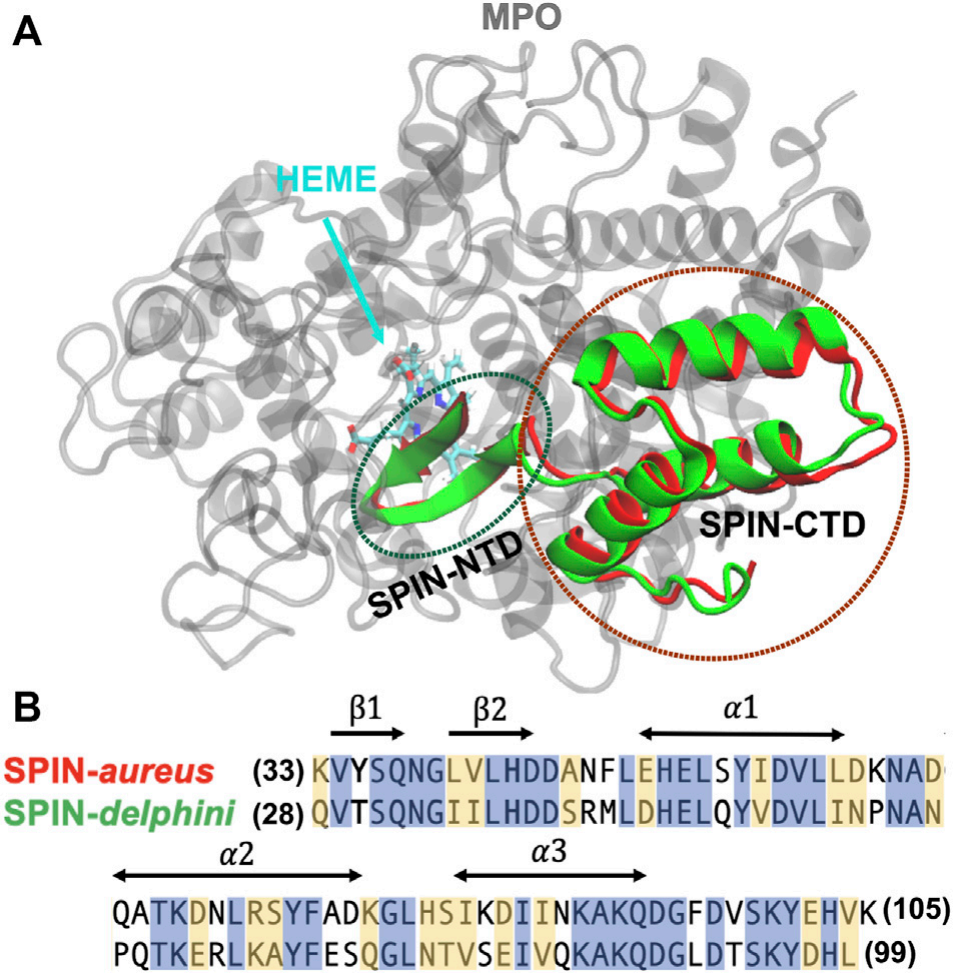
**(A)** Overlay of structures SPIN-*aureus* and SPIN-*delphini* in complex with human MPO. The structures were taken from PDB 5UZU and 6BMT for SPIN-*aureus* and SPIN-*delphini*, respectively. MPO is colored grey and SPIN-*aureus* and SPIN-*delphini* are colored red and green, respectively. Heme was shown in sticks. **(B)** NTD sequences of two SPIN homologs with conserved residues are highlighted in blue and similar residues in yellow. The sequence alignment is calculated using BLAST (Park et al., 2012) that shows 53% identity and 80% similarity. The secondary structures in the bound state are marked with arrows.

All simulations were performed with the GPU accelerated CHARMM/OpenMM interface (Brooks et al., 2009; Lee et al., 2016; Eastman et al., 2017) in CHARMM36m force field (Huang et al., 2017), which is one of the latest general-purpose protein force field specifically optimized for both IDPs and structured proteins. The initial structures of SPIN-*aureus* and SPIN-*delphini* in complex with human MPO were taken from the crystal structures [PDB 5UZU SPIN-*aureus* (de Jong et al., 2018) and 6BMT for SPIN-*delphini* (Ploscariu et al., 2018)] (see Figure 1A). To reduce the computational cost, only segments of MPO that are within 12 Å of SPIN are included in the current simulations, which consist of residues 167–200, 255–444, 490–506, 526–540, and 566–596 for MPO (Supplemetary Figure S1). To prevent the unfolding of MPO, all backbone heavy atoms of structured MPO segments (excluding loop residues 268–288, 380–395, and 317–328) and the bound heme group were restrained by harmonic potentials with a force constant of 1.0 kcal/(mol Å^2^) in all simulations. Proper amount of Na^+^ and Cl^−^ ions were added to neutralize the systems and to reach a NaCl concentration of 50 mM in accord with the experimental conditions (Ploscariu et al., 2018). The final solvated box contains about ∼30,000 TIP3P water molecules and has a dimension of ∼9.2nm^3^ × 9.4nm^3^ × 11.3 nm^3^.

Each solvated system was first energetically minimized for 500 steps using steepest decent and another 500 steps using the adopted basis Newton-Raphson algorithm. The system was then slowly heated up from 100 K to 300 K in 10 ps under the constant volume condition. Equilibration simulations were then performed at 300 K and 1 atm for a total of 1 ns, during which all protein heavy atoms were additionally restrained using harmonic potentials with force constants slowly decreasing from 5.0 kcal/(mol Å^2^) to 0.0 kcal/(mol Å^2^). Langevin thermostat was used to control the temperature and Monte Carlo barostat with volume move attempt every 25 steps was used to control the pressure. Lengths of all bonds involving hydrogen atoms were constrained using the SHAKE algorithm (Ryckaert et al., 1977) to allow for an integration time step of 2 fs. Long-range electrostatic interactions were treated using the particle mesh Ewald method (Darden et al., 1993), and the short-range van der Waals (vdW) interactions were treated with the twin-range cutoff at 12 and 14 Å.

To identify the optimal temperatures for unbinding/unfolding simulations, a series of pilot simulations were performed at temperatures ranging from 400 K to 500 K at 1 atm. Note that the complex was found to be highly stable below 400 K. Once an optimal temperature was identified (450 K), two sets of simulations were performed for each complex to probe temperature-induced SPIN unfolding and unbinding process. In one set, three additional simulations were performed at 450 K with different initial velocities to better characterize the dissociation of SPIN from MPO. These simulations were run until the NTD dissociated from the active pocket (i.e., with the fraction of native contacts between two molecules *Q*_inter_ < 0.3), which all occurred within 400 ns. In the second set, 40 independent replicas were performed for each complex at 450 K for 250 ns each, with the helical region of SPIN CTD (Figure 1B) harmonically restrained with a force constant of 1.0 kcal/(mol Å^2^). The purpose of the second set is to directly examine the unfolding and unbinding of the NTDs.

### Room temperature simulations of free NTDs

The initial folded hairpin structures of SPIN-NTDs were taken from the same complex structures (Figure 1A). Both systems contain a 13-residue fragment (SPIN-*aureus* residues 33–45 and SPIN-*delphini* residues 28–40; see Figure 1B for sequences). 20 replicas were used to simulate the unfolding events for two SPIN-NTDs at 300 K. The solvated systems contain ∼3,500 TIP3P waters and have dimensions of ∼4.2nm^3^ × 4.3nm^3^ × 5.4 nm^3^. Similar protocols as described above were applied to minimize and equilibrate the system. For each system, 20 independent production simulations were performed for 50 ns each at 300 K, which was sufficient to observe spontaneous unfolding of the β-hairpin structure.

### Analysis

All the analyses were carried out using CHARMM and additional in-house scripts. All molecular visualizations were prepared using VMD (Humphrey et al., 1996). The fractions of intermolecular and intramolecular native contacts, *Q*_inter_ and *Q*_intra_, are calculated to monitor the unfolding and unbinding process. The native contacts are first identified from the crystal structure of two complexes if the minimum heavy atom distance between two residues is no greater than 4.2 Å (Supplemetary Tables S1, S2). Note that for intramolecular native contacts, we exclude residue pairs that are close in sequence space and only consider those whose residue IDs are different by at least three. The contacts in simulation trajectories were then calculated using the same criterion. Based on protein folding funnel theory, native interactions dominate the overall pathway (Onuchic et al., 1996; Tsai et al., 1999; Shoemaker et al., 2000; Best et al., 2013). Therefore, only native contacts were considered here. The unbinding and unfolding kinetics were analyzed using a double exponential approximation of the decay of *Q*_inter_ and *Q*_intra_ averaged over all replica runs (40 for the complexes and 20 for free NTDs). The first 50 ns trajectories were considered in unfolding and unbinding kinetic analysis, which were sufficient to capture the dissociation events. Pseudo free energy surfaces were also calculated to better characterize the baseline mechanisms of coupled binding and folding, derived directly from two-dimensional (2D) probability distributions along *Q*_inter_ and *Q*_intra_. For the data used to construct contact probabilities, we specifically focused on short segments of the trajectories where actual dissociation transitions occurred. For example, only the first 15 ns trajectory in replica one of SPIN-*aureus*/MPO simulation was considered, which included the entire unbinding and unfolding transition (see Supplemetary Figure S2. By doing this, the results will not be interfered by the transient refolding events observed after complete dissociation (see Supplemetary Figure S2 replica 40 at 200 ns for example). The segments for each trajectory that were selected to calculate the contact maps can be found in Supplemetary Table S3. Note that for replicas where NTD remains bound and folded at the end of the 250 ns-simulation, we only selected the first 50 ns of trajectories to compute contact maps. In this way, we could avoid masking important details about the transition pathways by over-representing data of the bound and folded state.

## Results and discussion

### High temperature simulations reveal step-wise binding of SPIN NTD and CTD

**FIGURE 2:**
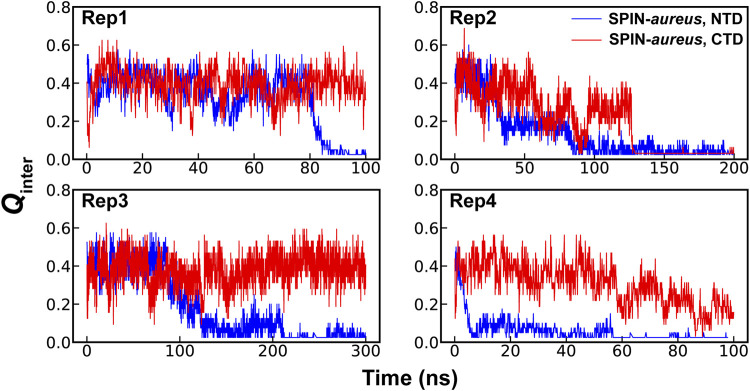
Fractions of native intermolecular contacts between the NTD (blue) and CTD (red) of SPIN during four independent simulations of the SPIN-*aureus*/MPO complex at 450 K.

High temperature simulations have been shown to be capable of providing reliable mechanistic insights in to folding of structured proteins as well as coupled binding and folding of IDPs (Verkhivker et al., 2003; Daggett, 2006; Chen and Luo, 2007; Schaeffer et al., 2008; Zhang et al., 2012). The assumption here is that unfolding and unbinding is largely a reverse of coupled binding and folding. While many mechanistic details derived from high-temperature simulations have compared well to experiments (Verkhivker et al., 2003; Daggett, 2006; Chen and Luo, 2007; Schaeffer et al., 2008; Zhang et al., 2012), it is also known that the most probable transition pathways may depend on the temperature (Dinner and Karplus, 1999). Therefore, it is important to find the lowest temperature to drive the unfolding and unbinding process within a given simulation timeframe. The pilot simulations suggest that the NTD of SPIN-*aureus* only starts to dissociate from the active pocket of MPO at 450 K within ∼100 ns timescale, which becomes much faster at higher temperatures (Supplemetary Figure S1B). Note that rapid dissociation (e.g., at 475 K) is not always preferred due to the risks of missing important details under non-physiological conditions and activating pathways not generally accessible under the physiological conditions. For example, the three-helix bundle of SPIN CTD would melt rapidly at 475 K and above, leading to premature disassociation from MPO within 10 s of ns. This is consistent with the experimental observation that SPIN CTD largely dictates MPO binding (de Jong et al., 2018). Instead, simulations at 450 K seem to depict a more realistic dissociation process, where NTD unbinds first while the CTD remains largely fold and bound (Figure 2). The apparent decoupling and step-wise nature of the binding of SPIN CTD and NTD could explain why there is little correlation between the inhibition strength and binding affinity for different SPIN homologs. It’s likely that two domains of SPIN bind and function almost independently when interacting with MPO. As such, some SPIN homologs, e.g., SPIN-*agnetis*, show comparable nanomolar binding affinity as SPIN-*aureus*, but have no detectable inhibitory ability to human MPO (Ploscariu et al., 2018). Based on these observations, we will focus on the coupled binding and folding of SPIN NTD while the CTD is harmonically restrained to the bound state in subsequent simulation and analysis.

### Cooperative binding and folding of SPIN-*aureus* NTD

**FIGURE 3:**
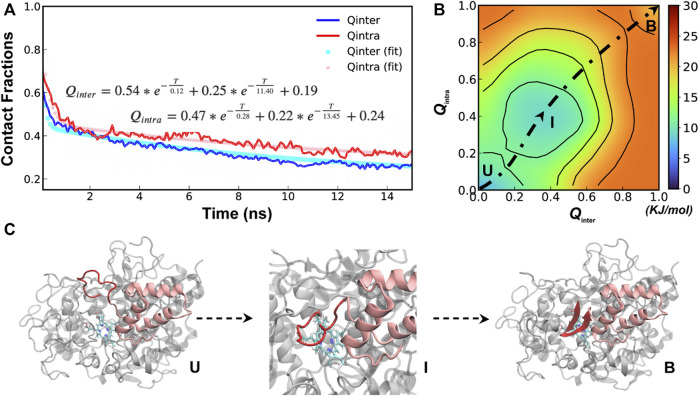
Cooperative binding and folding of SPIN-*aureus* NTD. **(A)** Average intramolecular and intermolecular native contact fractions (*Q*_inter_ and *Q*_intra_) as a function of simulation time at 450 K. The double exponential fits are plot using dotted lines, with the actual parameters also shown. **(B)** Pseudo free energy surface as a function of *Q*_inter_ and *Q*_intra_ derived from the transition paths (see Methods). The dashed line indicates the minimum free energy pathway. Key states (U, I and B) are also labeled. **(C)** Representative conformations for key states along the minimum free energy path, with SPIN-*aureus* and MPO shown in red and light grey, respectively.

A total of 40 independent 250-ns simulations were performed at 450 K to explore the conformational fluctuations, dynamic interactions and dissociation pathways of SPIN-*aureus* NTD with human MPO. As summarized in Supplemetary Figure S2, SPIN-*aureus* NTD tends to dissociate rapidly and its unfolding and unbinding often happen simultaneously. For example, in 36 out of 40 replicas (except for replicas 6, 8, 16 and 25), NTD fully dissociated (with *Q*_inter_ < 0.2) within 200 ns. Particularly, among 30 out of the 36 runs (except for replicas 10, 11, 17, 23, 29 and 35) unbind/unfold occurred within the first 50 ns, or sometimes even more rapidly within 15 ns. To quantitively describe the dissociation process and probe the mechanisms of coupled binding and folding, we calculated the average fractions of intermolecular and intramolecular native contacts formed by NTD, denoted *Q*_inter_ and *Q*_intra_, respectively, from all replicas. The results were then fitted with a double exponential function (Figure 3A). Not surprisingly, the unbinding and unfolding kinetics of SPIN-*aureus* NTD are similar, consistent with the observation that they appear highly correlated. As shown in Figure 3A, the initial fast phase $\tau_{1}$ for unbinding and unfolding are 0.12 and 0.28 ns, respectively, followed by a slow phase unbinding ( $\tau_{2}$ of 11.40 ns) and unfolding ( $\tau_{2}$ of 13.45 ns). We further constructed the pseudo 2D free energy surface as a function of NTD *Q*_inter_ and *Q*_intra_, derived from the dissociation transition segments (see Methods for details). The result, shown in Figure 3B, confirms a highly cooperative mechanism of SPIN-*aureus* NTD coupled binding and folding with NTD *Q*_inter_ and *Q*_intra_ increasing simultaneously in a highly correlated fashion. The minimum free energy path (dashed line) largely follows the diagonal line expected for an ideally cooperative mechanism.

The free energy surface also reveals three major conformational states of NTD folding and binding to MPO. State B (bound), with both *Q*_inter_ and *Q*_intra_ above 0.8, is the fully folded and bound state, and State U (unbound), with both *Q*_inter_ and *Q*_intra_ below 0.2, is the fully unfolded and unbound state. In addition, there is a partially bound and folded substate, I (intermediate state), where the values of *Q*_inter_ and *Q*_intra_ are around 0.4. Representative conformations for the three states of the complex (Figure 3C) illustrate that SPIN-*aureus* NTD does not tend to pre-fold into some “native-like” β-hairpins conformations prior to binding to the active site of MPO, and *vice versa*. The cooperative nature of SPIN-*aureus* NTD is in contrast to previous experimental and computational studies of coupled binding and folding of IDPs into non-β-hairpin structures (Chen, 2012), where induced folding-like mechanisms are prevalent. However, this may not be surprising given the cooperative nature of folding of isolated β-hairpins (Munoz et al., 1997; Klimov and Thirumalai, 2000; Chen et al., 2018). In particular, the “speed-limit” of β-hairpin folding usually is ∼μs^−1^, much slower compared to helix-coil transitions (∼100 ns), due to the requirement of forming long-range interactions and the presence of entropy-dominant barriers. Therefore, once SPIN-*aureus* CTD is tightly bound, native-like interactions with the MPO surface play a direct role to facilitate the rapid folding of NTD and achieve a facile blockage of the MPO active site for inhibition.

### Conformational selection-like mechanism for SPIN-*delphini* NTD

Compared to SPIN-*aureus*, which is secreted by *S. aureus* that appears to be particularly adapted to survive the neutrophil-mediated immunity with the highest binding affinity (K_D_ = 15.9 nM) and inhibition strength (IC_50_ = 4.6 nM) to human MPO, SPIN-*delphini* has a moderate binding affinity (K_D_ = 310 nM) but the second strong inhibitory ability (IC_50_ = 29.7 nM) among nine SPIN homologs previously analyzed (Ploscariu et al., 2018). A possible explanation is that SPIN-*delphini* NTD may have evolved to be less dependent on the tight binding of CTD. Interestingly, high-temperature simulations indeed reveal significant differences between coupled binding and folding of NTDs from SPIN-*aureus* and SPIN-*delphini*. As summarized in Supplemetary Figure S3, ∼40% of the 40 (17/40) replicas failed to observe full dissociation of SPIN-*delphini* NTD’s during the 250 ns simulations, which is about 3-fold of ∼10% for SPIN-*aureus*. The implication is that SPIN-*delphini* NTD fits the active site of MPO tighter than SPIN-aureus NTD, which would be consistent with disproportionally strong inhibitory function of SPIN-*delphini* despite weakened overall binding affinity.

**FIGURE 4:**
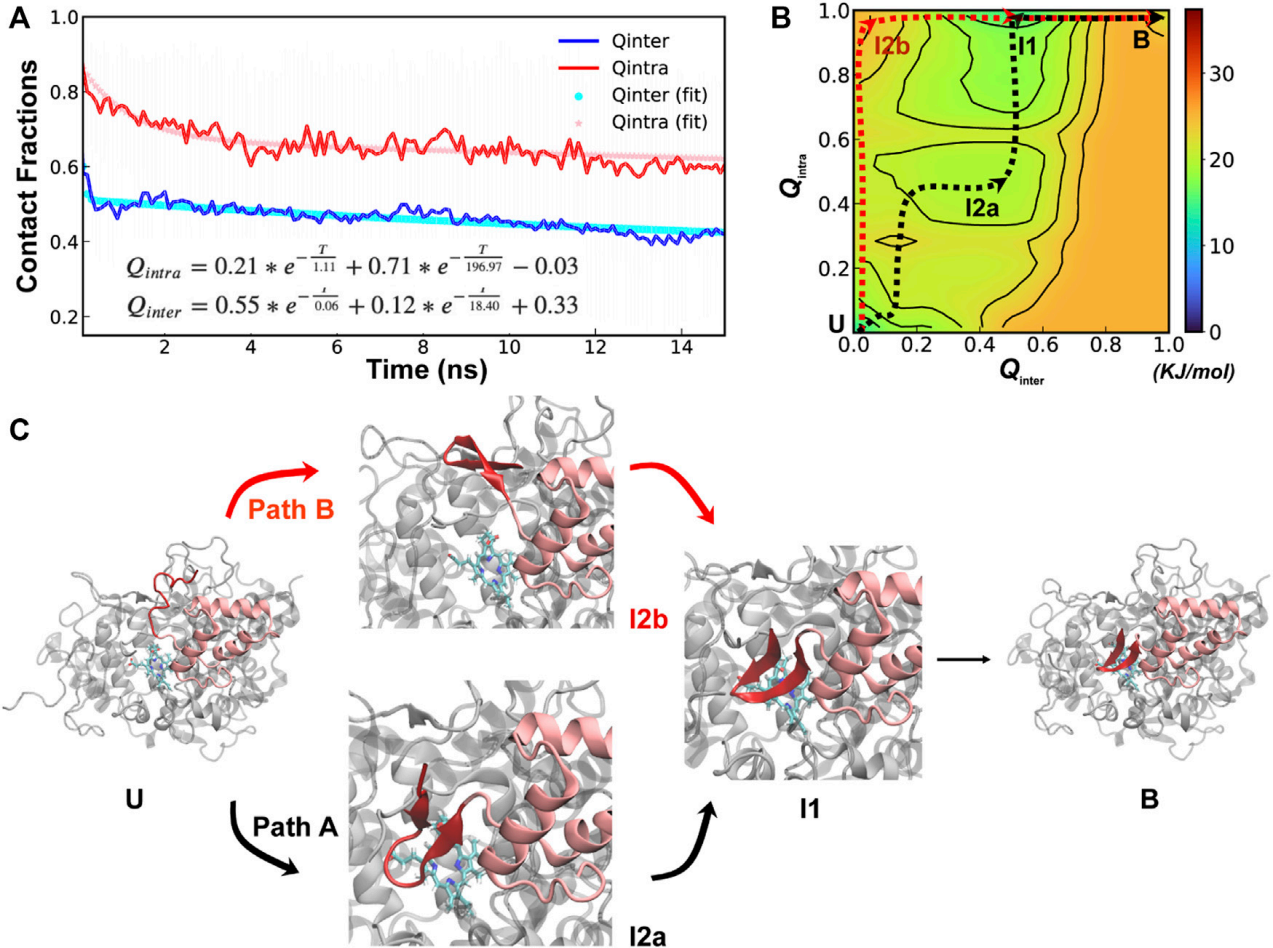
Cooperative binding and folding of SPIN-*delphini*. **(A)** Average intramolecular and intermolecular native contact fractions (*Q*_inter_ and *Q*_intra_) as a function of simulation time at 450 K. The double exponential fits are plot using dotted lines, with the actual parameters also shown. **(B)** Pseudo free energy surface as a function of *Q*_inter_ and *Q*_intra_ derived from the transition paths (see Methods). The dashed lines indicate the minimum free energy pathways, with key states labeled. **(C)** Two major parallel dissociation pathways and key intermediate states for coupled binding and folding of SPIN-*delphini* NTD to human MPO, with SPIN and MPO shown in red and light grey, respectively.

Further analysis of unfolding and unbinding kinetics and free energy surface reveal that SPIN-*delphini* NTD mainly follow a distinct conformational selection-like mechanism (Figure 4), where the NTD tends to gain substantial native β-hairpin structures prior to forming intermolecular interactions with MPO. This is well reflected in unbinding and unfolding kinetics. On average, the unbinding rates of SPIN-*delphini* NTD (( $\tau_{1}$ = 0.06 ns, $\tau_{2}$ = 18.4 ns, Figure 4A) are similar to those of SPIN-*aureus* NTD ( $\tau_{1}$ = 0.12 ns, $\tau_{2}$ = 11.4 ns, Figure 3A). However, the unfolding rates of SPIN-*delphini* NTD ( $\tau_{1}$ = 1.31 ns, $\tau_{2}$ = 196.97 ns) are over 10-fold slower than unbinding rates. In addition, SPIN-*delphini* NTD is considerably more folded at 15 ns, with *Q*_intra_ ∼ 0.6 compared to ∼0.3 for SPIN-*aureus* NTD. That is, while SPIN-*aureus* NTD unbinds and unfolds to similar levels at a given time (Figure 3A), SPIN-*delphini* NTD tends to retain much higher residual structures while it unbinds.

The minimal free energy paths, indicated by the dash lines in Figure 4B, demonstrate that SPIN-*delphini* NTD coupled binding and folding is not cooperative and follows two major routes with multiple intermediate states. Both routes go through an intermediate state I1, where there *Q*_inter_ drops below 0.7 while the β-hairpin structure is essentially intact with *Q*_intra_ ∼ 1.0. Overlay of representative structures from B (fully bound) and I1, shown in Supplemetary Figure S4, illustrates that how SPIN-*delphini* NTD becomes mobile within the active site pocket of MPO without unfolding. From state I1, the major pathway (path A) goes through another intermediate state I2a, which mainly has similar level of residual intermolecular native contacts (*Q*_inter_ ∼ 0.5) but the hairpin conformation becomes partially unfolded (*Q*_intra_ ∼ 0.5). From state I2a, SPIN-*delphini* NTD would further unbind and then unfold to reach the fully disassociated state (U). In the parallel pathway B, SPIN-delphini NTD would continue to become fully unbound from MPO without significant unfolding (I2b, *Q*_intra_ > 0.8, *Q*_inter_ < 0.2), before unfold outside of the MPO active site. The observed conformational selection-like mechanism of SPIN-*delphini* NTD interaction with MPO is summarized in Figure 4C. It shows that the disordered segment could become fully folded before inserting into the MPO active site (Path B), which is an ideally conformational selection mechanism. Such a process is best represented by high-temperature simulation run 24 (Supplemetary Figure S3, Rep24). Path A, which is more prevalent, involves multi-step conformational selections. In each step (U to I2a to B), the NTD first fold and then bind to MPO. The later pathway is best illustrated in Supplemetary Figure S3 Re33. The distinct mechanisms of SPIN-*aureus* and SPIN-*delphini* NTD coupled binding and folding may help explain why the inhibition strength doesn’t fully correlate with binding affinity among different SPIN homologs.

### Elevated pre-folding in unbound SPIN-*delphini* NTD

**FIGURE 5:**
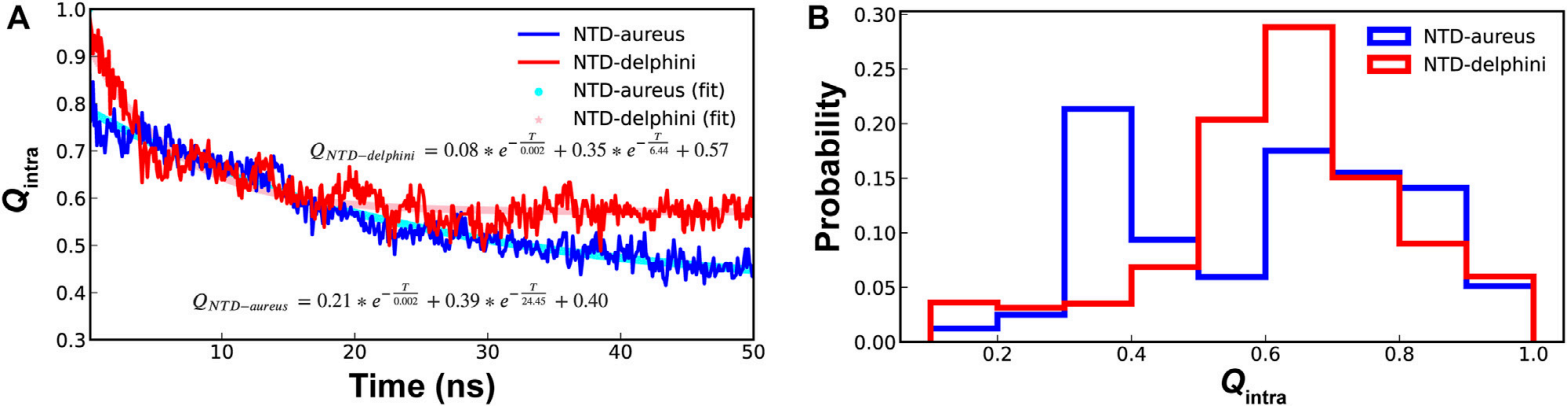
**(A)** Decay of NTD *Q*_intra_ for SPIN-aureus (blue) and SPIN-delphini (red) at 300 K starting with the fully folded conformation, averaged over 20 replicas of 50-ns simulations. **(B)** Distributions of NTD *Q*_*i*ntra_ for two SPIN homologs.

For conformational selection to be an efficient mechanism for coupled binding and folding, there should be high levels residual structures in unbound IDPs (Liu et al., 2019). Since SPIN-*aureus* NTD follows cooperative binding and folding while SPIN-*delphini* NTD prefers a conformational selection-like mechanism, we further characterized the stability of hairpin-like structures in their unbound states under the physiological conditions. As shown in Figure 5, although SPIN-*delphini* NTD showed slightly faster unfolding rates, it remained more structured than SPIN-*aureus* NTD. The limiting NTD *Q*_intra_ decayed to 0.57 and 0.40 for SPIN-*delphini* and SPIN-*aureus*, respectively (see Figure 5A). Importantly, the probability distributions of *Q*_intra_ show that there is a very high probability for SPIN-*delphini* NTD to remain partially folded (*Q*_intra_ > 0.5). Such an elevated residual β-hairpin structures in unbound SPIN-*delphini* NTD is consistent with the observation of conformational selection-like mechanism of its coupled binding and folding (see above). The more dynamic nature of SPIN-*aureus* NTD suggests that it depends on specific MPO interactions to facilitate its folding into the β-hairpin structure, thus following a cooperative binding and folding mechanism (Figure 3B).

## Conclusion

Extensive atomistic simulations have been performed in explicit solvent to gain a deeper understanding of the structural basis of how SPIN, a protein secreted by *Staphylococcus*, inhibits the activity of human MPO to help evade the neutrophil-mediated host innate immunity. It has been shown that the folded SPIN CTD can bind to MPO even in the absence of the disordered NTD, but the latter is required for the MPO inhibition function. Structural studies further revealed that SPIN NTDs folded into similar β-hairpins upon binding and inserted into the MPO active site for inhibition. Curiously, there is a poor correlation between the MPO binding affinity and inhibition efficacy among different SPIN analogs. The implication is that the conformational properties of unbound SPIN NTDs and their coupled binding and folding likely play central roles in their MPO inhibitory activity.

To further address these questions, we carried out extensive atomistic simulations in explicit solvent using the CHARMM36m force field and studied the structures and interactions of two SPIN homologs, namely, SPIN-*aureus* and SPIN-*delphini*. At an optimal temperature of 450 K, high-temperature simulations reveal that SPIN CTD and NTD binding to MPO follows a decoupled step-wise mechanism, consistent with the experimental observation that CTD is mainly responsible for specific MPO binding (de Jong et al., 2018). Further 450 K simulations of the unbinding and unfolding of SPIN NTD with CTD restrained in the bound state revealed striking difference in SPIN-*aureus* and SPIN-*delphini*. While coupled binding and folding SPIN-aureus NTD during interaction with MPO is highly cooperative, that of SPIN-*delphini* mainly follows a conformational selection-like mechanism. Both are in contrast to a prevalence of induced folding-like mechanism previously observed in experimental and computational studies of IDPs that fold into relatively simple structures such as helices and ordered loops (Chen, 2012). This is an important new insight on coupled binding and folding of IDPs that is likely applicable to other IDPs that require the formation of long-range interactions for specific binding. Non-etheless, we caution that not all mechanistic details generated at high temperature will be true at room or physiological temperature. Additional experimental and/or computational studies will be required to further validate the above predictions. For example, steered MD approaches (Lu and Schulten, 1999) could provide a viable alternative that may introduce less perturbation to the unfolding/unbinding pathway. A caveat is to find an appropriate pulling speed that is gentle enough but computationally feasible.

Atomistic simulations at room temperature further reveal that the mechanistic difference between SPIN-*aureus* and SPIN-*delphini* may be related to the intrinsic conformational properties of their NTDs in the unbound state. Specifically, SPIN-*aureus* NTD is more dynamic and less structured, requiring MPO binding to facilitate its folding and thus a cooperative binding and folding mechanism. On the other hand, SPIN-*delphini* NTD has a much higher propensity to adopt pre-folded hairpin-like conformations, allowing it to follow a conformational selection-like mechanism. As such, SPIN-*delphini* NTD is less dependent on CTD binding to MPO for specific interaction and MPO inhibition. These structural and mechanistic differences could explain why SPIN-*delphini* binds to MPO ∼19 times weaker than SPIN-*aureus*, but its IC_50_ is only ∼6 times higher. Taken together, the current atomistic simulations do not only provide new mechanistic principles on coupled binding and folding of IDPs into non-trivial β-hairpins, but also help to establish the structure-dynamics-function relationship of SPIN homologs. Moreover, it may suggest a new strategy to combating *Staphylococcus* infection, such as by designing drug molecules that could destabilize residual structures in SPIN NTD.

## Data Availability

The original contributions presented in the study are included in the article/Supplementary Material, further inquiries can be directed to the corresponding author.
