# Supplementary material for: Coupled binding and folding of disordered SPIN N-terminal region in myeloperoxidase inhibition
Source: Front Mol Biosci. 2023 Feb 9;10:1130189. doi: 10.3389/fmolb.2023.1130189 (PMC9948029; doi:10.3389/fmolb.2023.1130189)
Supplement: Supplementary file 1 [file DataSheet1.PDF]

Coupled binding and folding of disordered SPIN N-terminal region in myeloperoxidase inhibition

Yumeng Zhang, Xiaorong Liu[#] and Jianhan Chen*

[1]Department of Chemistry
University of Massachusetts
Amherst, MA 01003, USA

* Corresponding Authors: Email: jianhanc@umass.edu (JC), Phone: (413) 545-3386 (JC)

[#] Current Address: Department of Chemistry, University of Michigan, Ann Arbor, MI 48109

Yumeng Zhang and Xiaorong Liu makes equal contribution

Table S1. Native intermolecular contacts between SPIN and MPO, including the SPIN-*aureus* in complex with MPO (col 1st-4th) and SPIN-*delphini* in complex with MPO (col 5th-8th). The intermolecular native contacts between SPIN-NTD and MPO are highlighted in grey.

SPIN-*aureus*	MPO	SPIN-*aureus*	MPO	SPIN-*delphini*	MPO	SPIN-*delphini*	MPO
33	192	47	381	30	265	42	381
33	346	47	383	30	404	42	382
34	270	48	381	31	268	42	383
34	272	49	352	31	269	43	381
35	265	49	380	31	270	44	352
35	346	49	381	32	265	44	379
35	404	49	400	32	266	44	380
36	268	50	368	32	268	44	381
36	269	50	379	33	268	44	400
36	270	51	352	33	282	45	368
37	265	51	364	33	313	46	352
37	266	51	365	33	573	46	364
37	268	51	367	33	577	46	378
37	405	51	378	33	581	46	400
38	268	51	400	33	586	47	352
38	313	52	352	34	268	50	354
38	577	54	367	34	282	50	355
38	586	55	354	34	313	50	356
39	271	55	355	35	386	50	357
39	282	55	356	35	532	62	359
39	313	55	357	35	573	63	356
40	532	68	357	35	576	63	357
40	576	71	359	35	577	67	356
40	577	72	356	36	270	70	352
41	382	72	357	36	382	70	356
41	386	75	351	36	386	89	381
42	380	75	352	37	265	91	381

42	382	75	356	37	380
42	404	79	352	37	382
42	405	94	381	37	404
42	532	94	383	37	405
43	349	96	381	37	532
43	380			38	349
43	382			38	380
43	400			38	382
43	404			38	400
44	272			38	404
44	382			39	382
45	349			40	348
45	352			40	352

Table S2. Native intramolecular contacts within NTDs of SPIN-*aureus* and SPIN-*delphini*.

SPIN-*aureus* (NTD)	SPIN-*delphini* (NTD)
33 - 43	29 - 36
33 - 44	29 - 38
33 - 45	29 - 39
34 - 41	30 - 36
34 - 43	30 - 37
34 - 44	30 - 38
35 - 40	31 - 35
35 - 41	31 - 36
35 - 42	32 - 37
35 - 43	
35 - 45	
36 - 40	
37 - 42	

Table S3. Representative dissociation transition segments used in contact probability calculations for SPIN-*aureus*/MPO (left) and SPIN-*delphini*/MPO (right) selected from the whole 250 ns trajectories. Cases where NTD remains bound and folded at the end of simulations are colored in blue, where only the first 50 ns are selected for contact map calculations.

	**SPIN-*aureus*/MPO**	**SPIN-*delphini*/MPO**
0-15 ns	Rep 1,4,5,7,9,13,14,15,18,21,22,24,27,28,30,31,32,33,34,36,37,38,40	
0-25 ns		Rep 2,4,5,6,8,20,23,26,31
0-50 ns	Rep 2,3,6,8,12,16,19,20,25,26,39	Rep 3,7,9,10,11,12,13,14,15,16,18,20,21,22,25,27,28,29,32,34,37,38,39,40
0-100 ns	Rep 10,11,17,23,29,35	
0-80 ns		Rep 1,17,33,35
0-150 ns		Rep 19,24,36

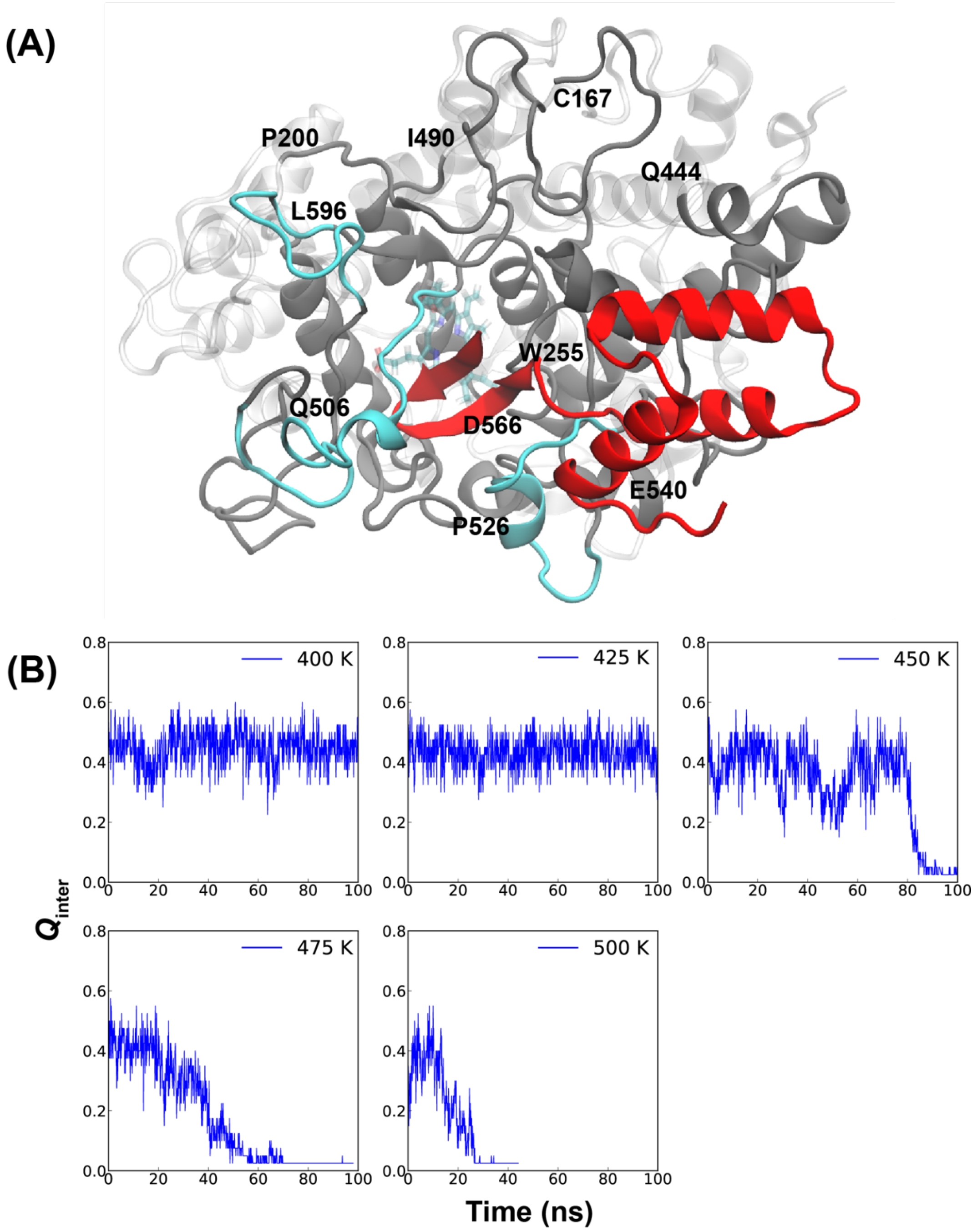

Figure S1. (A) Truncated SPIN/MPO complex system, with included regions colored in grey opaquely and omitted parts colored transparently for MPO. SPIN is colored in red. The MPO loop regions that are not restrained during simulations are colored in cyan. And HEME (not contained in the truncated system) is colored by name and drawn by Bonds. The terminal residues of the selected segments of MPO were labeled out. (B) Dissociation of SPIN-NTD measured by Q_{inter} during pilot of simulations at different temperatures from 400 K to 500 K.

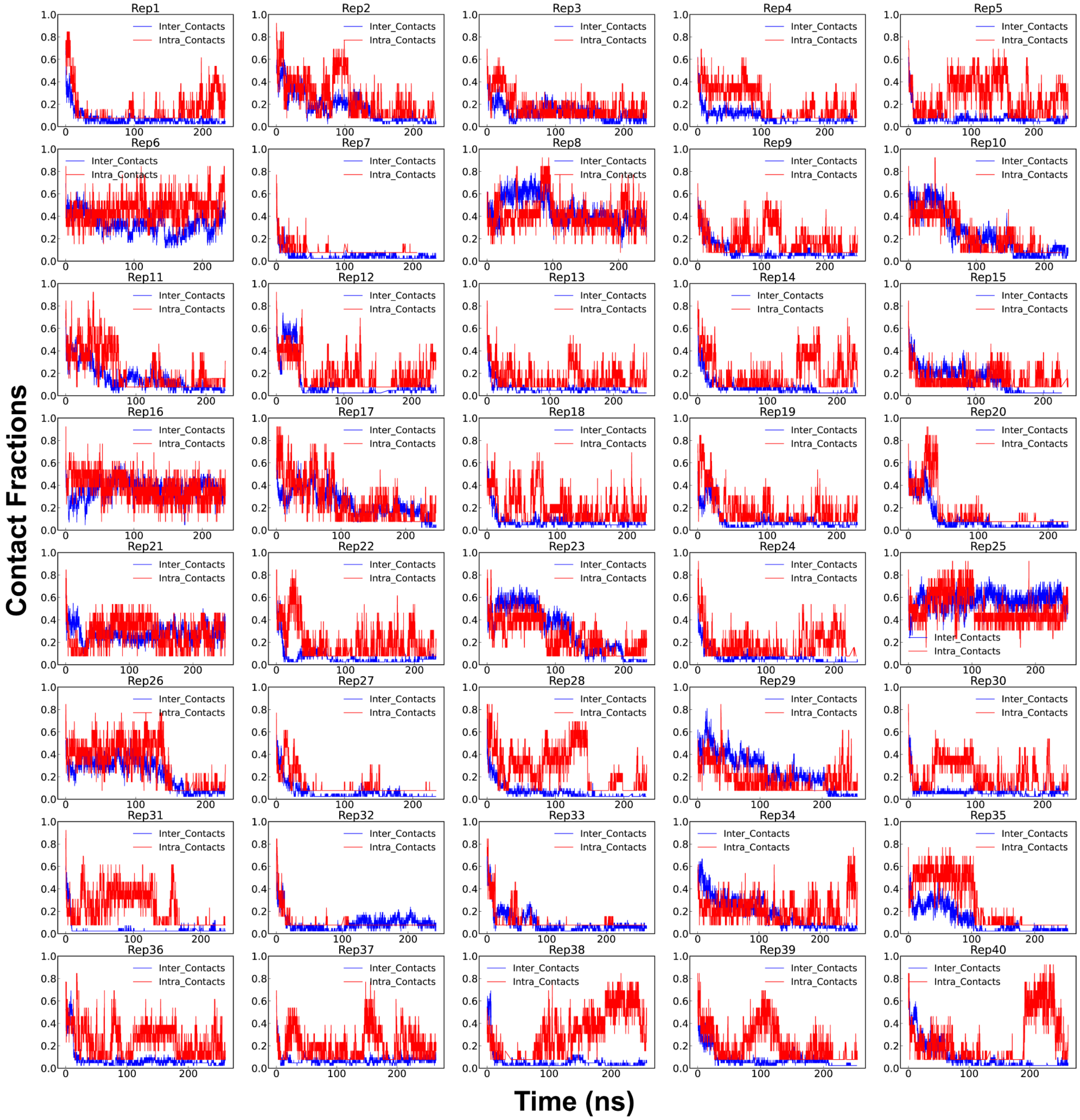

Figure S2. Time evolution of NTD Q_{inter} (blue) and Q_{intra} (red) native contact fractions during 40 replicas of 250 ns simulations of the SPIN-*aureus*/MPO complex at 450 K.

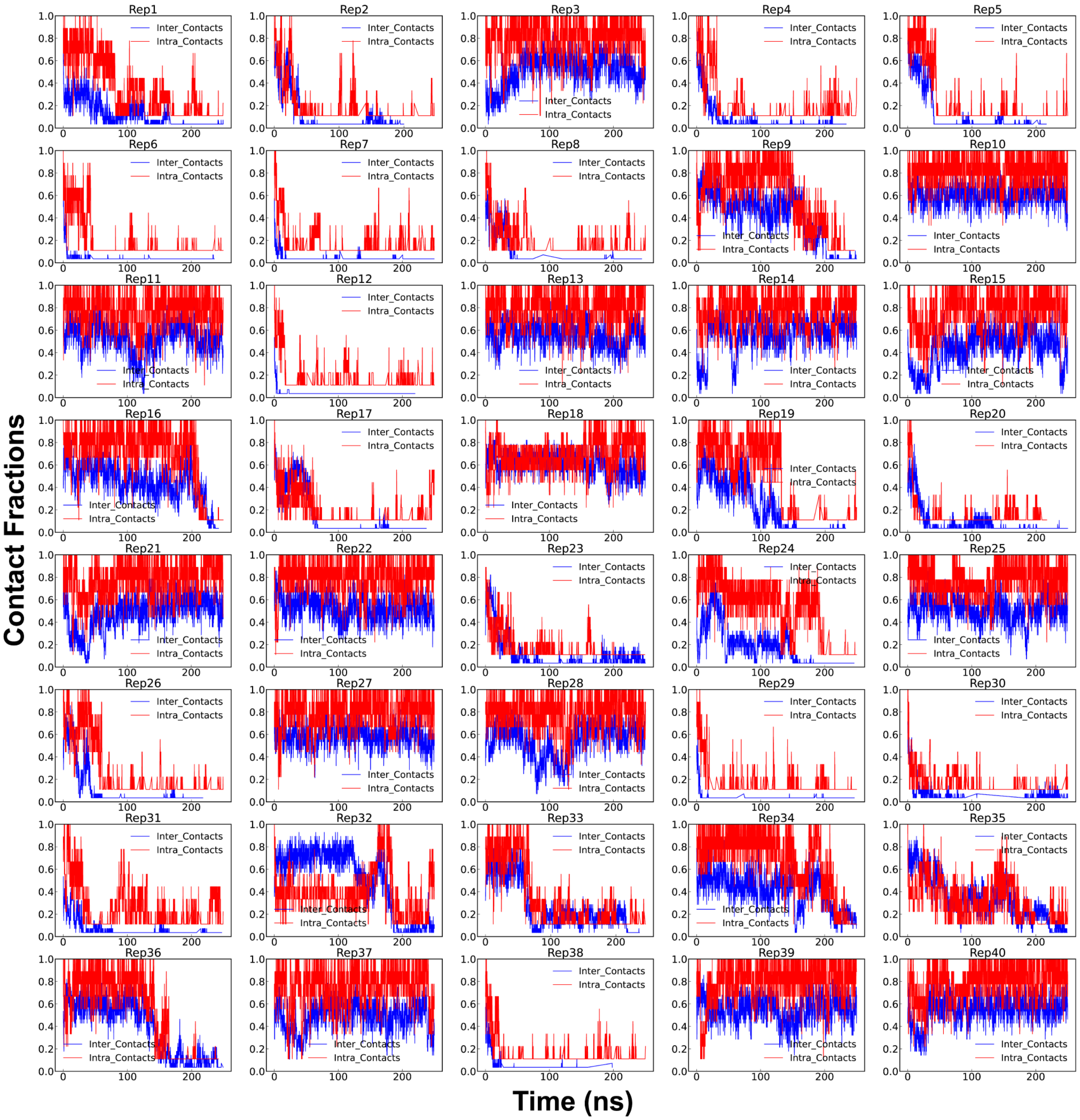

Figure S3. Time evolution of NTD Q_{inter} (blue) and Q_{intra} (red) native contact fractions during 40 replicas of 250 ns simulations of the SPIN-*delphini*/MPO complex at 450 K.

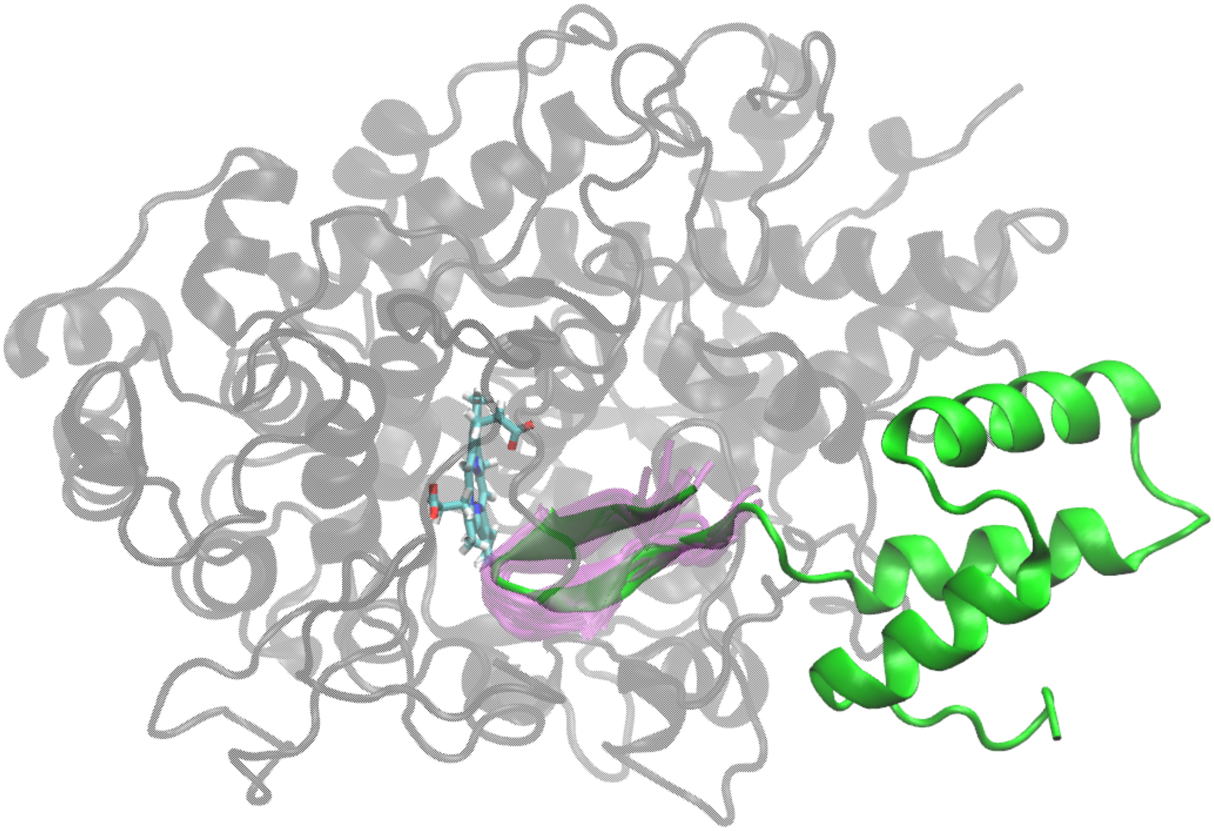

Figure S4. Overlay of the structures of SPIN-*delphini* in complex with MPO (State I1, Q_{inter} = 1.0, Q_{inter} ~0.6), with NTD ensemble transparently drawn (purple) compared to the initial bound and fold structure (green, explicitly drawn). MPO is colored grey and heme is shown in sticks.
